# Intraspecific Genetic Variation of *Anisakis typica* in Indian Mackerel Caught from the Gulf of Thailand, Samut Songkhram Province

**DOI:** 10.1155/2022/2122619

**Published:** 2022-06-21

**Authors:** Tanawat Chaiphongpachara, Poom Adisakwattana, Nantana Suwandittakul

**Affiliations:** ^1^Department of Public Health and Health Promotion, College of Allied Health Sciences, Suan Sunandha Rajabhat University, Bangkok, Thailand; ^2^Department of Helminthology, Faculty of Tropical Medicine, Mahidol University, Bangkok, Thailand

## Abstract

*Anisakis* nematodes infecting Indian mackerel (*Rastrelliger kanagurta*) were initially discovered in Thailand in our preliminary investigation. Nevertheless, the species of *Anisakis* collected has not been determined nor has its genetic variation been researched. Thus, this study aimed to molecularly identify the species of *Anisakis* specimens using the internal transcribed spacer (ITS) region of ribosomal DNA sequences. In addition, the intraspecific genetic variation was also determined using mitochondrial cytochrome oxidase subunit II (COII) gene sequences. The phylogenetic relationships of the ITS region classified all samples into *Anisakis typica*; however, the genetic variation between them could not be distinguished. By contrast, the phylogenetic tree analysis of the COII region identified all samples as *A*. *typica*, with 17 different haplotypes by 66 polymorphic sites and five of the substitutions resulted in amino acid change. Additionally, the distribution pattern of the COII region can be separated into two groups between South America and Asian countries. All our haplotypes belong to Asian countries. Compared with the two genetic markers used in this investigation, COII appears to be a better candidate for studying genetic variation sensitive to environmental changes and intermediate or definitive host behavioral changes.

## 1. Introduction

Anisakids are gastrointestinal fish-borne parasitic nematodes causing anisakiasis, first reported in the 1960s in the Netherlands [1]. More than 20,000 cases were reported worldwide every year [2]. These nematodes are parasites in the genus *Anisakis*, *Pseudoterranova*, and *Contracaecum* belonging to the phylum Nemathelminthes, class Nematoda, order Ascarida, and family Anisakidae [3]. According to previous findings, parasites in the genus *Anisakis*, particularly *Anisakis simplex* (sensu lato) complex, and *A. pegreffii* most frequently infect and cause diseases in humans [4, 5]. Because raw fish infected with third-stage larvae (L3) of *Anisakis* spp. is the transmission mode, a high prevalence of anisakiasis has been observed in countries where raw seafood is consumed culturally, including other countries worldwide [5]. Anisakiasis causes several mostly acute symptoms occurring within 12 h of ingesting the raw or semiraw fish and squid or marine products in patients due to allergic reactions and direct tissue damage from the penetration of the infective larvae [6].

The distribution of *Anisakis* spp. is related to several factors, such as global warming, oceanographic conditions, water circulation, salinity percentages, and host density and migration [7]. Additionally, several species of marine fish can serve as intermediate or paratenic hosts for *Anisakis* spp., with the L3 encyst residing in internal tissues awaiting ingestion by marine mammals (definitive host) or humans (accidental host) [8]. Therefore, surveillance of *Anisakis* infection in marine fish can provide crucial information for preventing disease outbreaks by alerting seafood vendors and consumers of the risk [2, 9, 10].

Thailand is a peninsular country whose over 3,500 km territory is connected to coastlines. The first case of intestinal anisakiasis in Thailand was reported in 1993 [11]. However, additional investigation on *Anisakis* epidemiology in humans and marine fish has been restricted. Although Thailand is not an epidemic area, global warming and environmental changes may affect the infection and spread of several parasitic diseases, including anisakiasis [12]. In the previous surveillance, more than 80 species of marine fish in Thailand were examined for *Anisakis* infections and found that approximately seven marine fish species were infected with these parasites, including *Muraenesox* spp., *Epinephelus areolatus*, *Rachycentron canadum*, *Trichiurus lepturus* [13], *Priacanthus tayenus* [14], *Nemipterus hexodon*, and *Nemipterus japonicus* [2]. Furthermore, our recent data were the first to detect *Anisakis* infection in Indian mackerel (*Rastrelliger kanagurta*), a commercial fish in Southeast Asia [15]. However, species identification of those parasites obtained from Indian mackerel has not been done. Moreover, information regarding the genetic variation of *Anisakis* spp. in Thailand remains unclear.

The parasitic nematodes in the Anisakidae family are very similar and are known as the complex parasitic group that is difficult to distinguish by morphological techniques. Consequently, the most accurate methods of identifying *Anisakis* parasites are the molecular biological methods, including polymerase chain reaction (PCR), polymerase chain reaction–restriction fragment length polymorphism, and next-generation sequencing [7]. Presently, the internal transcribed spacer (ITS) region of ribosomal DNA (ITS-1, 5.8S rRNA gene, and ITS-2) and the mitochondrial cytochrome oxidase subunit II (COII) gene are considered and recognized as molecular markers for species identification and genetic variation of anisakid nematodes [2, 7, 14, 16].

This study utilized *Anisakis* larvae from Indian mackerel (*R. kanagurta*) for the DNA amplification and sequencing of the ITS region and COII gene. Additionally, phylogenetic trees were constructed to estimate the evolutionary relationships, which are crucial for understanding parasite population dynamics in the ecosystem and their hosts.

## 2. Materials and Methods

### 2.1. Ethics Statement

This study was performed strictly according to the guidelines of the Suan Sunandha Rajabhat University, Thailand, on animal care and use. All animal use protocols were reviewed and approved by the Suan Sunandha Rajabhat University' Institutional Animal Care and Use Committee (ref. LACUC 002/2021).

### 2.2. DNA Extraction

*Anisakis* specimens detected in a previous study in Indian mackerel (*R. kanagurta*) [15] were used for molecular analysis. Genomic DNA was extracted from whole individual *Anisakis* specimen using FavorPrep™ Tissue Genomic DNA Extraction Mini Kit (Favorgen Biotech, Ping-Tung, Taiwan), according to the manufacturer's instructions. The DNA concentration was measured using a Nanodrop One-c spectrophotometer (Thermo Fisher Scientific, Madison, WI).

### 2.3. DNA Amplification and Sequencing

The amplification of ITS regions (ITS-1, 5.8S subunit, ITS-2) and COII gene of *Anisakis* specimens was performed following previous publications with some modifications [17, 18]. The forward and reverse primers for amplification of ITS regions were NC5: 5′-GTA GGT GAA CCT GCG GAA GGA TCA TT-3′ and NC2: 5′-TTA GTT TCT TCC TCC GCT-3′, respectively. The forward and reverse primers for amplification of the COII gene were 211F: 5′-TTT TCT AGT TAT ATA GAT TGR TTT YAT-3′ and 211R: 5′-CAC CAA CTC TTA AAA TTA TC-3′, respectively [19]. The length of ITS (ITS-1, 5.8S subunit, and ITS-2) were 815 bp. By contrast, the length of COII was 591 bp. For ITS regions, the reaction mixture (50 *μ*L) comprised of 5 *μ*L of gDNA and 45 *μ*L of PCR mix containing 1x reaction buffer, 1.5 mM MgCl_2_, 200 *μ*M dNTPs, 0.25 *μ*M of each primer, 1.5 U of Taq polymerase, and the remaining volume of distilled water up to 45 *μ*L. PCR conditions included an initial denaturation step for 10 min at 95°C, followed by 30 cycles of 30 s at 95°C, 30 s at 55°C, 75 s at 72°C, and a final extension step of 7 min at 72°C. For COII gene, the reaction mixture (50 *μ*L) contained 5 *μ*L of gDNA and 45 *μ*L of PCR mix containing 1x reaction buffer, 2.5 mM MgCl_2_, 0.4 mM dNTPs, 0.3 *μ*M of each primer, 5 U of Taq polymerase, and the remaining volume of distilled water up to 45 *μ*L. The PCR conditions consisted of an initial denaturation step for 3 min at 94°C, followed by 34 cycles of 30 s at 94°C, 60 s at 46°C, 90 s at 72°C, and a final extension step of 10 min at 72°C. The PCR products were analyzed by 1.5% agarose gel electrophoresis prior to be submitted for DNA sequencing (SolGent Co., Ltd., Daejeon, Korea).

### 2.4. DNA Sequence Analysis

After nucleotide sequencing, the trace files/chromatograms of ITS and COII sequences were checked manually in BioEdit [20]. Both forward and reverse sequences in each sample were compared and aligned to generate a consensus sequence using CLUSTAL W [21] in BioEdit. Consensus sequences were then compared with previously published sequences in the GenBank database using the basic local alignment search tool (BLAST), freely available at https://blast.ncbi.nlm.nih.gov/Blast.cgi for species identification based on measuring sequence similarities [22, 23].

The sequences of ITS and COII of our samples and other related sequences recruited from the NCBI database (Supplementary Table 1) were obtained for the construction of phylogenetic trees using the maximum likelihood method (1000 bootstrap replication) of MEGA X [24]. Substitution models from the MEGA X software were tested before constructing maximum likelihood trees to determine the most appropriate analytical model. This study used the Kimura 2-parameter (K2P) model, and bootstrap analysis with 1000 replicates to build a maximum likelihood tree on the basis of the ITS region. The Tamura-Nei + gamma distribution, conversely, is well suited to create a maximum likelihood tree on the basis of the COII region. Overall mean intraspecific divergence of ITS and COII sequences was calculated to compare the variability of all *Anisakis* sequences using MEGA X. Conversely, the number of mutations, parsimony informative sites, haplotypes, genetic diversity indices (haplotype diversity and nucleotide diversity), and neutrality tests (Tajima's D and Fu's FS) were calculated using the DNA Sequences Polymorphism software version 6 [25]. Finally, the haplotype network was illustrated using PopArt 1.7 to evaluate the interrelationship among haplotypes.

## 3. Results

### 3.1. Molecular Identification

**Figure 1:**
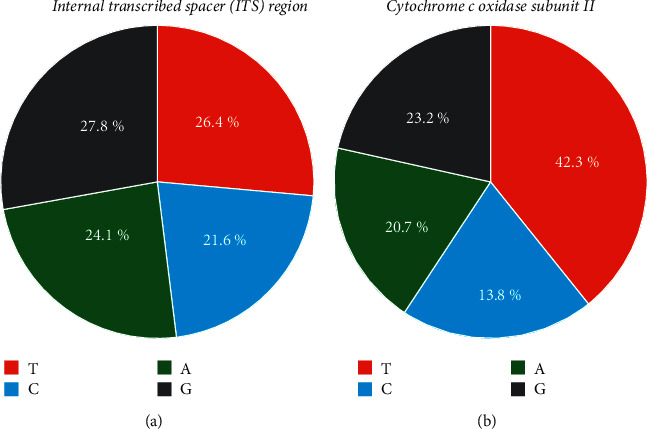
Overall average base composition of the ITS (ITS-1, 5.8S subunit, ITS-2) (a) and COII ((b)) fragments.

Seventeen *Anisakis* samples were amplified and further analyzed from 23 samples obtained in a previous study. By contrast, another six samples yielded negative results despite optimizing various conditions, indicating that they must be excluded. The overall average base composition of the ITS and COII fragments is shown in Figure 1. For COII, the AT content (adenine + thymine, 63%) was greater than the GC content (guanine + cytosine, 37%). Conversely, the ITS, AT, and GC contents were similar (50.5% vs. 49.5%). According to the sequence homology comparison with the GenBank database, the results indicated that both ITS and COII regions of all samples have high identity with *A*. *typica*. ITS regions showed 100% identity, whereas COII showed 97.77%–99.83% identity with *A*. *typica* available in the database (Table 1).

**Figure 2:**
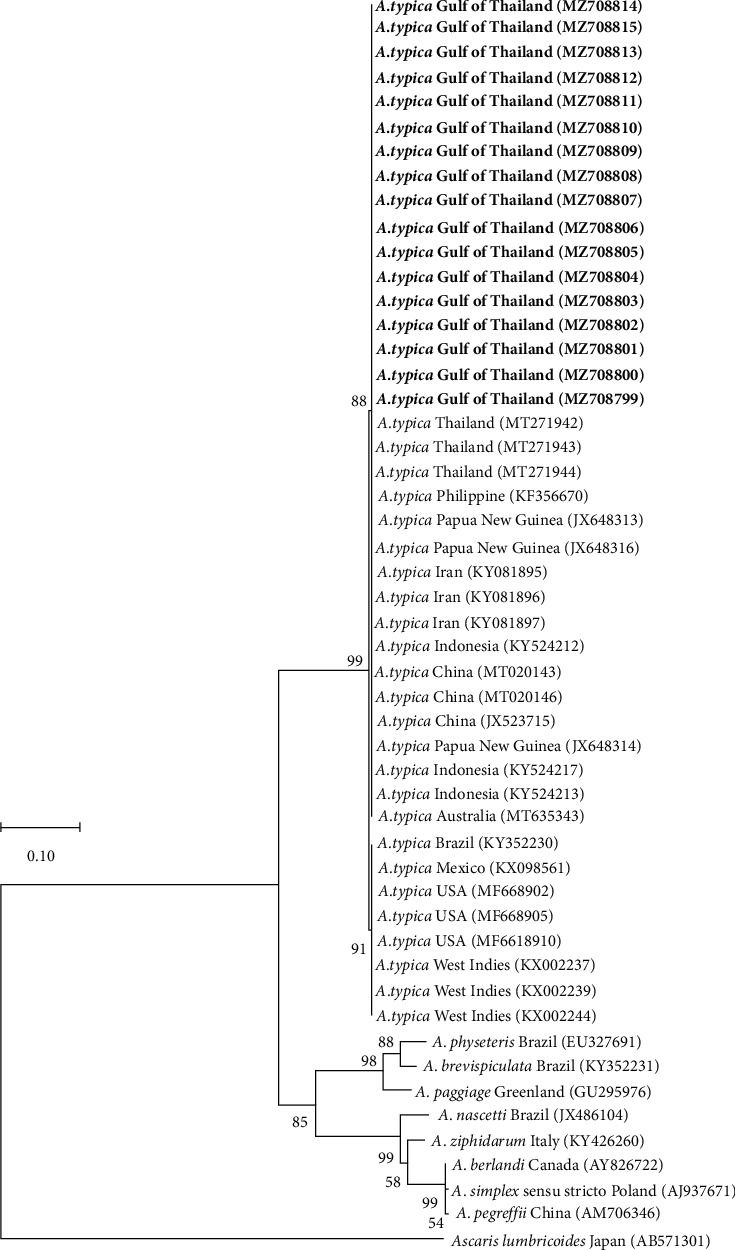
Maximum likelihood tree with 1000 bootstrap replicates, constructed on the basis of ITS region (ITS-1, 5.8S subunit, and ITS-2) of *A. typica* sequences from the Gulf of Thailand and other countries retrieved from GenBank. The bold font in this phylogenetic tree shows the samples of the present study. At the same time, one sequence of *Ascaris lumbricoides* was added for outgroup taxa.

The phylogenetic analysis of ITS indicated that all 17 samples were clustered with other *A. typica* reported in other studies. Although, *A. pegreffii* and *A. simplex* sequences retrieved from GenBank were separated from all *A. typica* sequences of both ITS, which strongly supported the *A. typica* clade. *Ascaris lumbricoides*, considered as outgroup taxa, were separated from the *Anisakis* genus. In addition, the ITS region was unable to differentiate *A. typica* found in this study genetically on the basis of geographical locations (Figure 2).

### 3.2. Intraspecific Genetic Variation

**Figure 3:**
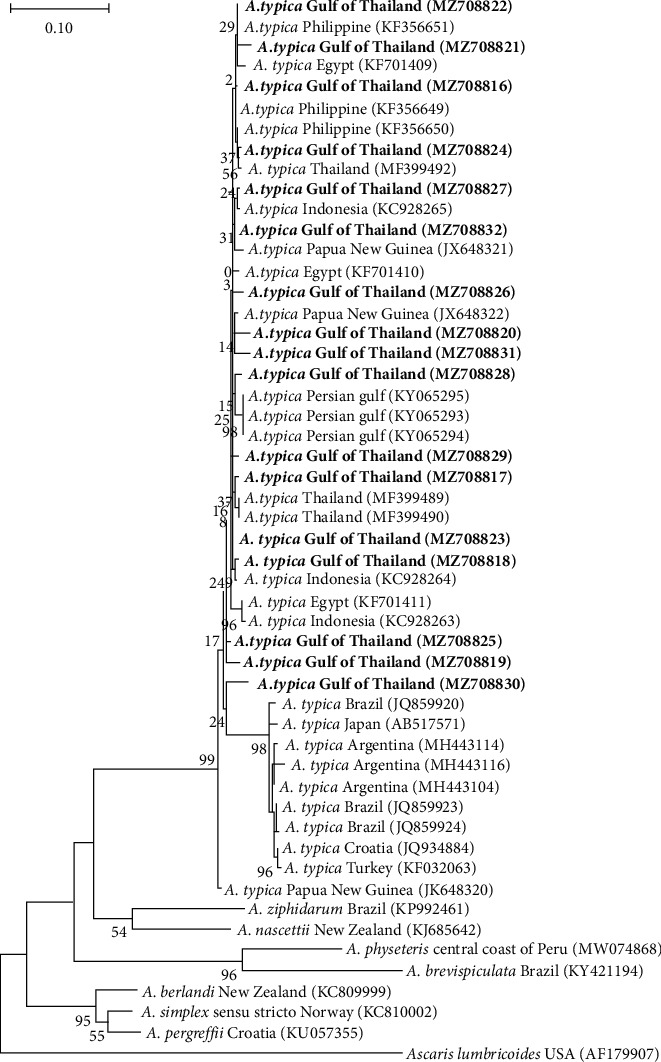
Maximum likelihood tree with 1000 bootstrap replicates, constructed on the basis of COII gene of *A. typica* sequences from the Gulf of Thailand and other countries retrieved from GenBank. The bold font in this phylogenetic tree shows the samples in the present study. At the same time, one sequence of *Ascaris lumbricoides* was added for outgroup taxa.

**Figure 4:**
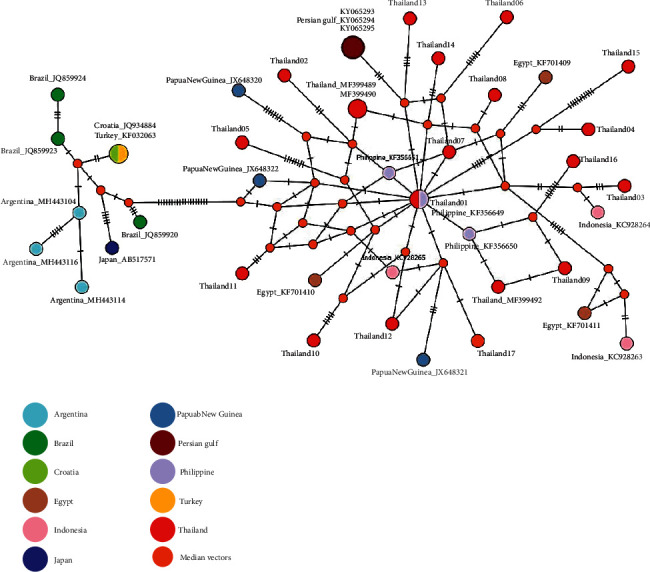
Haplotype network based on 44 COII sequences of *A*. *typica* from Thailand and other countries (GenBank accession numbers of each sequence are given in Supplementary Table 1). The size of the circle corresponds to the frequency of each haplotype. The hatch mark in each branch represents the number of mutations observed. Small orange circles indicate median vectors used in connecting indirectly related haplotypes.

Contrary to ITS, COII sequences from seventeen *A*. *typica* exhibited species identification and genetic variants with an average intraspecific divergence of 0.0206 (Table 2). The alignment of all COII sequences revealed 66 polymorphic sites (Supplementary Table 2), which had 41 singleton variable sites (62.12%) and 25 parsimony informative sites (37.88%). A total of 17 haplotypes and 68 mutations were identified. The number of amino acid substitution checked with five of the substitutions resulted in amino acid change including at a position 25 (S ⟶ R) in haplotype 16, at a position 100 (M ⟶ I) in haplotype 4, at a position 103 (V ⟶ I) in haplotype 16, at a position 154 (R ⟶ S) in haplotype 15, and at a position 156 (I ⟶ V) in haplotype 16. At the same time, the overall haplotype diversity and nucleotide diversity were 1.00 ± 0.020 and 0.0196 ± 0.0026, respectively. Tajima's D and Fu's FS statistics were negative but statistically not significant either (−1.779, *P* > 0.05 and −8.340, *P* > 0.05, respectively). The phylogenetic analysis of COII sequences indicated that *A. typica* divided into two clusters on the basis of geographical locations, including Asia Pacific and American regions (Figure 3). While, our COII gene haplotype network revealed 17 different haplotypes across our *A*. *typica* samples, with mutations discovered in several positions each (Figure 4).

## 4. Discussion

The Indian mackerel *R. kanagurta* (Cuvier, 1817) is a pelagic schooling fish widely distributed in the Indian Ocean and Indo-West Pacific region [26]. In several countries of Southeast Asia such as Indonesia, Malaysia, Myanmar, and Thailand, they are an economically important marine fish [27, 28]. In the past, the *Anisakis* parasite was found in Indian mackerel in some countries, including China [29], Indonesia [30, 31], and Malaysia [32] but has never been reported in Thailand. However, our latest study detected *Anisakis* infections in Indian mackerel fish from the Samut Songkhram fish market [15]. Food contaminated with pathogens is essential to planning for disease surveillance both of physical and genetical; even though anisakiasis is not a common disease in Thailand, there should not be complacency. Consequently, our study aimed at species identification of the *Anisakis* parasite and genetic variability in Indian mackerel from the Gulf of Thailand.

In the past, at least before molecular techniques, the species of *Anisakis* parasites was difficult to identify with a light microscope because of their similar morphology characteristics [33, 34]; thus, the details of *Anisakis* species were mysterious. Nowadays, however, molecular techniques act as tools to assist in identifying *Anisakis* species effectively with a gene that represents specific species. Generally, the ITS region of ribosomal DNA (ITS1, 5.8S rRNA gene, and ITS2) and the mitochondrial COII gene were widely used for identifying *Anisakis* species. Moreover, both genes have an advantage to differentiate phylogenetic trees as well [35].

In our investigation, molecular analysis using ITS and COII revealed that 17 *Anisakis* samples from Indian mackerels captured in Thailand are *A. typica* (Diesing, 1860, Nematoda: Anisakidae), also documented in several surveillances of Indian mackerels caught in Indonesia [30, 31]. These findings may indicate that Indian mackerels are an important intermediate host of this parasite in Southeast Asia. *Anisakis typica* has not yet been reported to cause disease in humans, although *A. simplex*, *A. physeteris*, and *A. pegreffii* are the most common human pathogens [36]. However, only a few human anisakiasis cases were investigated further at a species level since obtaining the parasite requires an invasive gastroduodenoscopy and biopsy forceps operation [37].

The analysis of the phylogenetic relationships of ITS and COII based on maximum likelihood trees revealed the grouping between our *Anisakis* sequences with *A. typica* sequences retrieved from GenBank. Additionally, the results of the comparison with the GenBank database by BLAST showed more than 98% best match from more than 50 *A*. *typica* sequences of both ITS region and COII gene. These results conclusively confirm that all of our samples were *A. typica*. In Thailand, there were definite reports based on molecular species identification of *A. typica* parasite detected in purple-spotted big eye fish (*Priacanthus tayenus*) with only one gene that is COII gene but not ITS region [14] and threadfin bream (*Nemipterus hexodon* and *N*. *japonicus*) with ITS region but not COII gene [2]. By contrast, our study applied both ITS and COII genes to confirm *Anisakis* species to each other. The investigation of nucleotide compositions of COII sequences of *A. typica* indicated high A + T richness (>60%), differing from nucleotide compositions of ITS sequences in which both A + T and G + C contents had a similar number, approximately 50%. These results are consistent with previous studies that found high A + T rich of the mitochondrial gene in several parasitic nematodes such as *Radopholus similis* [38] and *Toxocara* spp. [39] and found a similar number of A + T and G + C contents of ITS in many nematodes such as *Contracaecum osculatum* [40].

Our study found no genetic variation in ITS regions among our *A. typica* samples. Although there was no variation in our group, phylogenetic tree based on ITS region revealed our *A. typica* differed from the samples of the American region (including Brazil, Mexico, West Indies of North America, and the United States) but similar to the Asia-Pacific continent composed of China, Papua New Guinea, and Indonesia. The result of our ITS phylogenetic tree was consistent with a study on *A. typica* from threadfin breams in the Gulf of Thailand that found no genetic variation in this group as well (genetic distance = 0%), but a group separation between sequences from Asia, Europe, and America [2]. There are reasonably two possibilities for this phenomenon, including (1) genetic variations caused by intensely different environmental conditions and climate change that directly affect the definitive and intermediate hosts of both the life cycle and their behavior of the journey in the ocean or (2) the presence of the species complex. Nevertheless, the results of this study were insufficient to decide what they are or may occur with multifactorial. The verification required taking samples from both areas for further comparative analysis of several genes with the morphological study. Recently, Chan and his colleague demonstrated a novel way of analyzing genetic distances from several genes that correlated with species identification of helminths to generate suitable cutoff values per genetic marker for each taxonomic level using the “K-means” clustering algorithm [41]. Chan's study could be useful for further research with a phylogenetic analysis of *A*. *typica* with a selection of suitable and standardized genes compared to other samples worldwide [41].

Apart from that, the phylogenetic results of the COII gene showed genetic variation in all samples with high levels. An analysis of COII identified 17 distinct haplotypes, which were representative sequences of *A. typica* in the present study. These results were consistent with those of the Eamsobhana et al. report in which *A. typica* in purple-spotted big eye fish from the Gulf of Thailand reported high variation of COII by detecting nine haplotypes from a total of 15 samples [14]. The haplotype and nucleotide diversities of our result and Eamsobhana and his colleagues' study were close together by our study revealing the haplotype and nucleotide diversity at 1 ± 0.02 and 0.0196 ± 0.0026, respectively, while the study of Eamsobhana and coworker show the haplotype and nucleotide diversity at 0.9683 ± 0.0193 and 0.0336 ± 0.0171, respectively. Conversely, Tajima's D and Fu's FS test of neutrality values of our analysis showed negative, indicating an excess number of alleles and an excess low-frequency polymorphism in the populations but not statistically significant.

Following the results of COII variation found in Indian mackerel fish of the Gulf of Thailand with 17 haplotypes, geographical distributions were shown. However, on the phylogenetic tree (Figure 3), the distribution pattern can be separated into two groups between South America and Asian countries. Our haplotypes belong to Asian countries such as the Philippines, Indonesia, Papua New Guinea, and Egypt, whereas another group belongs to South America such as Argentina and Brazil. Understandably, this phenomenon is due to the definitive and intermediated hosts (Indian mackerel fish) that presented as a mirror of *A. typica* infection [42]. Generally, *A. typica* found in the definitive hosts such as the Delphinoidea superfamily mainly inhabits deep water in tropical and warm temperate waters where their food lived within these areas [43]. Consequently, the Indian mackerel fish is one of the paratenic hosts of the genus *Scombridae*. Thus, they have been mostly found in Indian Oceans, which correlated with each other according to the COII haplotype pattern in our sample. However, the study of Pekmezci and his colleagues [34] show the haplotypes from Turkey are found in the South American clade because there are *A. typica* from marine fishes collected in Turkish waters (Mediterranean Sea). The presence of *A. typica* in the eastern Mediterranean Sea (Aegean Sea) could be a consequence of the migration of paratenic or definitive hosts from the Indian Ocean to Mediterranean waters through the Suez Channel [35]. However, our COII haplotype patterns cannot be divided into a specific country as seen in linked patterns, possibly due to genetic variations through migration or travel between the intermediated fish host in Indian Oceans. Although, in some part of Japan, the haplotypes (AB517571, collected from *Scomber japonicus* marine fish) found on South America clade were possibly eaten by definitive host such as Delphinidae and taken far away; however, there are no clearly evidence. Additionally, we have not found that our 17 COII haplotype patterns linked to South American countries or Atlantic Ocean might be associated with long migration distances and habitat of Indian mackerel fish unsuitable for this area zone, including water temperature and changing tides.

Our analytical results showed that the ITS region of rDNA was less variable than the COII gene. Hence, this study indicated the ITS region as the appropriate genetic marker for *A. typica* larvae molecular identification. At the same time, the COII gene could be suitable for investigating genetic variations linked to different environments due to intraspecific genetic variability that occurs easily.

## 5. Conclusions

This study is a report from Thailand with Indian mackerel (an important popular fish dish) infected with *A. typica*. Nonetheless, *A. typica* infection in Thai people is quite rare because Indian mackerel is not commonly consumed raw. However, the current trend of eating raw seafood in Thailand is continuously emerging from the popularity of Japanese and Korean foods. Consequently, the results of this investigation will support dietary precautions, especially marine fish, which should not be consumed raw and must be properly cooked to prevent harm from this parasite. Additionally, this research promotes the molecular parasite species identification using the ITS region of rDNA more than the COII gene on the basis of intraspecific genetic variability. Moreover, because of intraspecific genetic variability, COII gene stability may be linked to different environment changes and intermediate and definitive host behavior. However, several factors affecting COII variations of *A. typica* should be addressed later.

## Figures and Tables

**Table 1:** Percent identity in the GenBank database, GenBank accession numbers, and number of nucleotide sequence of *Anisakis* samples.

Sample codes	ITS (*n* = 17)			COII (*n* = 17)		
	Identity (%) in the GenBank database	GenBank accession no.	No. of nucleotide sequence (bp)	Identity (%) in the GenBank database	GenBank accession no.	No. of nucleotide sequence (bp)
01	*A. typica* (100%)	MZ708799	815	*A. typica* (99.83%)	MZ708816	591
02	*A. typica* (100%)	MZ708800	815	*A. typica* (99.31%)	MZ708817	591
03	*A. typica* (100%)	MZ708801	815	*A. typica* (99.32%)	MZ708818	591
04	*A. typica* (100%)	MZ708802	815	*A. typica* (98.77%)	MZ708819	591
05	*A. typica* (100%)	MZ708803	815	*A. typica* (98.32%)	MZ708820	591
06	*A. typica* (100%)	MZ708804	815	*A. typica* (98.31%)	MZ708821	591
07	*A. typica* (100%)	MZ708805	815	*A. typica* (99.83%)	MZ708822	591
08	*A. typica* (100%)	MZ708806	815	*A. typica* (99.49%)	MZ708823	591
09	*A. typica* (100%)	MZ708807	815	*A. typica* (99.79%)	MZ708824	591
10	*A. typica* (100%)	MZ708808	815	*A. typica* (98.99%)	MZ708825	591
11	*A. typica* (100%)	MZ708809	815	*A. typica* (98.97%)	MZ708826	591
12	*A. typica* (100%)	MZ708810	815	*A. typica* (99.48%)	MZ708827	591
13	*A. typica* (100%)	MZ708811	815	*A. typica* (99.16%)	MZ708828	591
14	*A. typica* (100%)	MZ708812	815	*A. typica* (99.33%)	MZ708829	591
15	*A. typica* (100%)	MZ708813	815	*A. typica* (97.77%)	MZ708830	591
16	*A. typica* (100%)	MZ708814	815	*A. typica* (98.48%)	MZ708831	591
17	*A. typica* (100%)	MZ708815	815	*A. typica* (99.66%)	MZ708832	591

Percent identity in this table shows the value of the sequence with the highest in the GenBank database.

**Table 2:** Summary statistics of ITS region and COII gene diversity of *A. typica*.

*Anisakis typica*	ITS (*n* = 17)	COII (*n* = 17)
Overall average intraspecific divergence	0	0.0206
No. of mutations	0	66
Parsimony informative sites	0	25
No. of haplotypes	1	17
Haplotype diversity (Hd) ± SD	0	1.00 ± 0.020
Nucleotide diversity (*π*) ± SD	0	0.0196 ± 0.0026
Tajima's D	—	−1.779
Fu's Fs	—	−8.340

## Data Availability

DNA sequences of *A. typica* used to support this study are available in the GeneBank Database, accession numbers: MZ708799–MZ708832 (Table 1).

## Supplementary Materials

Supplementary Materials Supplementary Table 1: list of sequences used in phylogenetic analysis. Supplementary Table 2: nucleotide variation at 66 polymorphic sites in COII gene of *A. typica* in this study.
