# Supplementary material for: Intraspecific Genetic Variation of Anisakis typica in Indian Mackerel Caught from the Gulf of Thailand, Samut Songkhram Province
Source: ScientificWorldJournal. 2022 Jun 21;2022:2122619. doi: 10.1155/2022/2122619 (PMC9239807; doi:10.1155/2022/2122619)
Supplement: Supplementary Materials — Supplementary Table 1: list of sequences used in phylogenetic analysis. Supplementary Table 2: nucleotide variation at 66 polymorphic sites in COII gene of A. typica in this study. [file 2122619.f1.zip › 2122619.f1/Supplementary Table 1 (FL).docx]

**Supplementary Table 1:** List of sequences used in phylogenetic analysis

| **GenBank Accession no.** | ***Anisakis* spp. And** | **Countries** | **Sequence sources** |
| --- | --- | --- | --- |
| **1. The internal transcribed spacer (ITS) region** | | | |
| MZ708799-MZ708815 | 1. *typica* | Gulf of Thailand | This study |
| MT635343 | 1. *typica* | Australia | GenBank |
| KY352230 | 1. *typica* | Brazil | GenBank |
| JX523715 | 1. *typica* | China | GenBank |
| MT020143 | 1. *typica* | China | GenBank |
| MT020146 | 1. *typica* | China | GenBank |
| KY524212 | 1. *typica* | Indonesia | GenBank |
| KY524213 | 1. *typica* | Indonesia | GenBank |
| KY524217 | 1. *typica* | Indonesia | GenBank |
| KY081895 | 1. *typica* | Iran | GenBank |
| KY081896 | 1. *typica* | Iran | GenBank |
| KY081897 | 1. *typica* | Iran | GenBank |
| KX098561 | 1. *typica* | Mexico | GenBank |
| JX648313 | 1. *typica* | Papua New Guinea | GenBank |
| JX648314 | 1. *typica* | Papua New Guinea | GenBank |
| JX648316 | 1. *typica* | Papua New Guinea | GenBank |
| KF356670 | 1. *typica* | Philippine | GenBank |
| MT271942 | 1. *typica* | Thailand | GenBank |
| MT271943 | 1. *typica* | Thailand | GenBank |
| MT271944 | 1. *typica* | Thailand | GenBank |
| MF668902 | 1. *typica* | USA | GenBank |
| MF668905 | 1. *typica* | USA | GenBank |
| MF668910 | 1. *typica* | USA | GenBank |
| KX002237 | 1. *typica* | West Indies | GenBank |
| KX002239 | 1. *typica* | West Indies | GenBank |
| KX002244 | 1. *typica* | West Indies | GenBank |
| AJ937671 | *A. simplex sensu stricto* | Poland | GenBank |
| AM706346 | *A. pegreffii* | China | GenBank |
| AY826722 | *A. berlandi* | Canada | GenBank |
| KY426260 | *A. ziphidarum* | Italy | GenBank |
| JX486104 | *A. nascettii* | Brazil | GenBank |
| GU295976 | *A. paggiae* | Greenland | GenBank |
| EU327691 | *A. physeteris* | Brazil | GenBank |
| KY352231 | *A. brevispiculata* | Brazil | GenBank |
| AB571301 | *Ascaris lumbricoides* | Japan | GenBank |
| **2. Mitochondrial cytochrome oxidase subunit II (COII) gene** | | | |
| MZ708816-MZ708832 | *A. typica* | Gulf of Thailand | This study |
| MH443104 | *A. typica* | Argentina | GenBank |
| MH443114 | *A. typica* | Argentina | GenBank |
| MH443116 | *A. typica* | Argentina | GenBank |
| JQ859920 | *A. typica* | Brazil | GenBank |
| JQ859923 | *A. typica* | Brazil | GenBank |
| JQ859924 | *A. typica* | Brazil | GenBank |
| JQ934884 | *A. typica* | Croatia | GenBank |
| KF701409 | *A. typica* | Egypt | GenBank |
| KF701410 | *A. typica* | Egypt | GenBank |
| KF701411 | *A. typica* | Egypt | GenBank |
| KC928263 | *A. typica* | Indonesia | GenBank |
| KC928264 | *A. typica* | Indonesia | GenBank |
| KC928265 | *A. typica* | Indonesia | GenBank |
| AB517571 | *A. typica* | Japan | GenBank |
| JX648320 | *A. typica* | Papua New Guinea | GenBank |
| JX648321 | *A. typica* | Papua New Guinea | GenBank |
| JX648322 | *A. typica* | Papua New Guinea | GenBank |
| KY065293 | *A. typica* | Persian gulf | GenBank |
| KY065294 | *A. typica* | Persian gulf | GenBank |
| KY065295 | *A. typica* | Persian gulf | GenBank |
| KF356649 | *A. typica* | Philippine | GenBank |
| KF356650 | *A. typica* | Philippine | GenBank |
| KF356651 | *A. typica* | Philippine | GenBank |
| MF399489 | *A. typica* | Thailand | GenBank |
| MF399490 | *A. typica* | Thailand | GenBank |
| MF399492 | *A. typica* | Thailand | GenBank |
| KF032063 | *A. typica* | Turkey | GenBank |
| KC810002 | *A. simplex sensu stricto* | Norway | GenBank |
| KU057355 | *A. pegreffii* | Croatia | GenBank |
| KC809999 | *A. berlandi* | New Zealand | GenBank |
| KP992461 | *A. ziphidarum* | Brazil | GenBank |
| FJ685642 | *A. nascettii* | New Zealand | GenBank |
| MW074868 | *A. physeteris* | Central coast of Peru | GenBank |
| KY421194 | *A. brevispiculata* | Brazil | GenBank |
| AF179907 | *Ascaris lumbricoides* | USA | GenBank |
