# Supplementary material for: Intraspecific Genetic Variation of Anisakis typica in Indian Mackerel Caught from the Gulf of Thailand, Samut Songkhram Province
Source: ScientificWorldJournal. 2022 Jun 21;2022:2122619. doi: 10.1155/2022/2122619 (PMC9239807; doi:10.1155/2022/2122619)
Supplement: Supplementary Materials — Supplementary Table 1: list of sequences used in phylogenetic analysis. Supplementary Table 2: nucleotide variation at 66 polymorphic sites in COII gene of A. typica in this study. [file 2122619.f1.zip › 2122619.f1/Supplementary Table 2 (FL).docx]

**Supplementary Table 2:** Nucleotide variation at 66 polymorphic sites in COII gene of *A. typica* in this study.

| Sample codes | Nucleotide at position | | | | | | | | | | | | | | | | | | | | | |
| --- | --- | --- | --- | --- | --- | --- | --- | --- | --- | --- | --- | --- | --- | --- | --- | --- | --- | --- | --- | --- | --- | --- |
|  | 006 | 016 | 031 | 036 | 057 | 066 | 073 | 075 | 084 | 099 | 114 | 126 | 153 | 156 | 168 | 179 | 195 | 196 | 225 | 231 | 237 | 249 |
| *A. typica* 01 | T | T | T | **C** | G | T | A | C | T | T | G | T | G | C | T | T | T | C | T | T | C | T |
| *A. typica* 02 | T | T | T | T | G | T | A | C | T | T | G | T | G | C | T | T | T | C | T | T | C | T |
| *A. typica* 03 | T | T | T | T | **A** | T | A | C | T | **C** | G | T | G | C | T | T | T | C | **C** | **C** | C | T |
| *A. typica* 04 | **C** | **C** | T | T | G | T | A | C | T | T | G | T | G | C | **C** | T | T | C | T | T | C | T |
| *A. typica* 05 | T | T | T | T | G | T | A | C | T | T | **T** | **C** | G | C | T | T | T | C | T | T | **T** | T |
| *A. typica* 06 | T | T | T | T | G | T | A | C | T | T | G | T | G | C | T | **C** | **C** | C | T | T | C | T |
| *A. typica* 07 | T | T | T | T | G | T | A | C | T | T | G | T | G | C | T | **C** | T | C | T | T | C | T |
| *A. typica* 08 | T | T | T | T | G | T | A | C | T | T | G | T | G | C | T | T | T | C | T | T | C | T |
| *A. typica* 09 | T | T | T | T | G | T | A | C | **C** | T | G | T | G | C | T | T | T | C | T | T | C | T |
| *A. typica* 10 | T | T | T | T | G | T | A | C | T | T | G | T | G | C | **C** | T | T | **T** | T | T | **T** | T |
| *A. typica* 11 | T | T | T | T | G | **C** | A | **T** | T | T | **A** | T | G | C | T | T | T | C | T | T | C | T |
| *A. typica* 12 | T | **C** | T | T | G | T | A | C | T | T | G | T | G | **T** | T | T | T | **T** | T | T | C | T |
| *A. typica* 13 | T | T | T | T | G | T | A | C | T | T | G | T | G | C | T | T | T | C | T | T | C | T |
| *A. typica* 14 | T | T | T | T | G | T | A | C | T | T | G | T | G | C | T | T | T | C | T | T | C | T |
| *A. typica* 15 | T | T | **C** | T | G | T | A | **T** | T | T | G | T | **A** | **T** | **C** | T | T | C | T | T | C | **C** |
| *A. typica* 16 | T | T | T | T | G | T | **C** | C | **C** | T | G | T | G | C | T | T | T | C | T | T | **T** | T |
| *A. typica* 17 | T | T | T | T | G | T | A | C | T | T | G | T | G | **T** | T | T | T | C | T | T | C | T |

**Supplementary Table 2:** Nucleotide variation at 66 polymorphic sites in COII gene of *A. typica* in this study (cont.).

| Sample codes | Nucleotide at position (cont.) | | | | | | | | | | | | | | | | | | | | | |
| --- | --- | --- | --- | --- | --- | --- | --- | --- | --- | --- | --- | --- | --- | --- | --- | --- | --- | --- | --- | --- | --- | --- |
|  | 252 | 261 | 267 | 270 | 273 | 279 | 280 | 288 | 300 | 307 | 309 | 312 | 318 | 333 | 337 | 354 | 360 | 363 | 369 | 372 | 375 | 384 |
| *A. typica* 01 | A | G | T | T | C | T | C | T | G | G | T | T | A | C | C | T | T | T | T | C | C | T |
| *A. typica* 02 | A | G | T | T | C | T | C | T | G | G | T | T | A | C | C | T | T | T | T | C | **T** | **C** |
| *A. typica* 03 | A | G | T | T | C | T | C | T | G | G | T | T | A | C | C | T | T | T | T | C | C | T |
| *A. typica* 04 | A | G | T | T | C | T | C | T | **A** | G | **C** | **C** | A | C | C | T | T | T | **C** | **T** | C | T |
| *A. typica* 05 | A | G | T | T | **T** | T | C | T | G | G | T | T | A | C | C | **C** | T | T | **C** | C | C | **C** |
| *A. typica* 06 | **G** | G | T | T | C | **C** | **T** | T | G | G | T | T | A | C | **T** | T | **C** | **A** | T | C | C | T |
| *A. typica* 07 | A | G | T | T | C | T | C | T | G | G | T | T | A | C | C | T | T | T | T | C | C | T |
| *A. typica* 08 | A | G | T | T | C | T | C | **C** | G | G | T | T | A | C | C | T | T | T | T | C | C | T |
| *A. typica* 09 | A | G | T | T | C | T | C | T | G | G | T | T | A | C | C | T | T | T | T | C | C | T |
| *A. typica* 10 | A | G | T | T | C | T | C | T | G | G | **C** | T | **G** | C | C | T | T | T | T | **T** | C | T |
| *A. typica* 11 | A | G | T | T | C | T | C | T | G | G | T | T | A | C | C | T | T | T | T | C | C | T |
| *A. typica* 12 | A | G | T | T | C | T | C | T | G | G | T | T | A | C | C | T | T | T | T | C | C | T |
| *A. typica* 13 | **G** | G | T | **C** | C | T | C | T | G | G | T | T | A | C | C | T | T | T | T | **T** | C | T |
| *A. typica* 14 | A | G | **C** | T | C | T | C | T | G | G | T | T | A | C | C | T | T | T | T | C | **T** | T |
| *A. typica* 15 | A | **A** | T | T | C | T | C | T | G | G | **C** | T | A | **T** | C | T | T | T | **C** | **T** | C | T |
| *A. typica* 16 | A | G | T | T | C | T | **T** | T | G | **A** | T | T | A | C | C | **C** | T | T | T | C | C | T |
| *A. typica* 17 | A | G | T | T | C | T | C | T | G | G | T | T | A | C | C | T | T | T | T | C | C | T |

**Supplementary Table 2:** Nucleotide variation at 66 polymorphic sites in COII gene of *A. typica* in this study (cont.).

| Sample codes | Nucleotide at position (cont.) | | | | | | | | | | | | | | | | | | | | | |
| --- | --- | --- | --- | --- | --- | --- | --- | --- | --- | --- | --- | --- | --- | --- | --- | --- | --- | --- | --- | --- | --- | --- |
|  | 390 | 393 | 402 | 408 | 414 | 417 | 432 | 435 | 456 | 462 | 466 | 471 | 480 | 507 | 513 | 519 | 522 | 534 | 540 | 543 | 546 | 558 |
| *A. typica* 01 | C | C | G | G | T | C | C | T | C | G | A | T | A | T | C | A | T | T | T | **T** | C | T |
| *A. typica* 02 | C | C | G | G | T | C | C | T | C | G | A | **C** | A | **C** | C | A | T | T | T | C | C | T |
| *A. typica* 03 | C | C | G | G | T | C | **T** | T | **T** | G | A | T | A | T | C | A | T | T | T | C | C | T |
| *A. typica* 04 | C | C | G | G | T | C | C | T | C | G | A | T | A | T | **T** | A | T | T | T | C | C | **C** |
| *A. typica* 05 | **T** | **T** | G | G | **C** | C | C | T | C | G | A | T | A | T | C | A | T | T | T | C | C | T |
| *A. typica* 06 | C | C | G | G | T | C | C | T | C | G | A | T | A | T | C | A | T | **C** | **C** | C | C | T |
| *A. typica* 07 | C | C | G | G | T | C | C | T | C | G | A | T | A | T | C | A | T | T | T | C | C | T |
| *A. typica* 08 | C | C | G | G | T | C | **T** | T | **T** | G | A | T | A | T | C | A | T | T | T | C | C | T |
| *A. typica* 09 | C | C | **A** | G | T | C | C | T | C | G | A | T | **G** | T | C | A | T | T | T | C | C | T |
| *A. typica* 10 | C | C | G | G | T | C | C | T | **T** | G | A | T | A | T | C | A | T | T | T | C | C | T |
| *A. typica* 11 | C | C | G | **T** | T | **T** | C | **C** | C | G | A | T | A | T | C | A | T | T | T | **T** | C | T |
| *A. typica* 12 | C | C | G | G | T | C | C | T | C | G | A | T | A | T | C | A | T | T | T | **T** | C | T |
| *A. typica* 13 | **T** | C | G | G | T | C | C | T | C | G | A | T | A | T | C | A | **C** | T | T | C | C | T |
| *A. typica* 14 | **T** | C | G | G | T | C | **T** | T | C | G | A | T | A | T | C | A | T | T | T | C | C | T |
| *A. typica* 15 | **T** | **T** | G | **T** | T | C | **T** | T | C | **T** | A | T | A | T | **T** | A | T | T | T | C | **T** | T |
| *A. typica* 16 | **T** | C | G | G | T | C | C | T | C | G | **G** | T | **G** | T | C | A | T | T | **C** | C | C | T |
| *A. typica* 17 | C | C | G | G | T | C | C | T | C | G | A | T | A | T | C | **G** | T | T | T | C | C | T |
